# Protein glycosylation and glycation in Alzheimer's disease: mechanisms, crosstalk, and therapeutic perspectives

**DOI:** 10.3389/fnins.2026.1925934

**Published:** 2026-10-08

**Authors:** Yuqi Luo, Jingjing Gu

**Affiliations:** School of Basic Medical Science, Zhejiang Chinese Medical University, Hangzhou, Zhejiang, China

**Keywords:** advanced glycation end products (AGEs), Alzheimer's disease, amyloid-β, biomarkers, protein glycosylation, tau hyperphosphorylation

## Abstract

Alzheimer's disease (AD) is the most prevalent neurodegenerative disorder worldwide, pathologically characterized by extracellular senile plaques composed of amyloid-β (Aβ) peptides and intracellular neurofibrillary tangles (NFTs) formed by hyperphosphorylated tau. Despite decades of research, the molecular mechanisms underlying AD pathogenesis remain incompletely understood, and effective disease-modifying therapies are still lacking. Protein glycosylation and glycation, two distinct post-translational modifications, have emerged as critical contributors to AD pathophysiology. Glycosylation, an enzymatically regulated process including N-linked glycosylation, O-GlcNAcylation, and O-GalNAc glycosylation, modulates the processing of amyloid precursor protein (APP), the aggregation propensity and hyperphosphorylation of tau, the function of apolipoprotein E (ApoE), and neuroinflammatory signaling. Aberrant glycosylation patterns have been consistently detected in the brain, cerebrospinal fluid (CSF), and plasma of AD patients, highlighting their potential as diagnostic biomarkers. In contrast, glycation is a non-enzymatic reaction between reducing sugars and proteins that generates advanced glycation end products (AGEs), which promote oxidative stress, neuroinflammation, blood-brain barrier (BBB) dysfunction, and Aβ and tau aggregation through interaction with the receptor for AGEs (RAGE). This review systematically summarizes recent advances in understanding the roles of glycosylation and glycation in AD pathogenesis, their crosstalk with classical hallmarks, and their translational value as biomarkers and therapeutic targets, providing novel perspectives for early diagnosis and multi-target intervention strategies for AD.

## 1 Introduction

Alzheimer's disease (AD) is a neurodegenerative disorder clinically characterized by progressive memory loss, cognitive decline, behavioral changes, and impaired activities of daily living (Soria Lopez et al., 2019). It is the leading cause of dementia in the elderly; as the disease progresses, memory and cognitive functions gradually deteriorate until patients ultimately lose the ability to care for themselves (Montgomery et al., 2018). Death typically occurs from 8 to 10 years after the initial diagnosis (Musicco et al., 2009). As of 2021, approximately 55 million people worldwide were affected by AD (D'Cruz and Banerjee, 2021), and this prevalence is projected to reach approximately 152 million by 2040 (Janoutová et al., 2021). In China, the number of AD cases by 2040 is expected to equal the combined total of all developed countries. The classical pathological hallmarks of the disease include extracellular senile plaques formed by the aberrant cleavage and aggregation of amyloid β-peptide (Aβ), intracellular neurofibrillary tangles (NFTs) composed of hyperphosphorylated tau, as well as accompanying synaptic loss and persistent neuroinflammation (Polanco et al., 2018; Rawat et al., 2022; Ma et al., 2022; Naseri et al., 2019). To date, however, the exact pathogenesis of AD remains elusive, and disease-modifying therapies are still lacking.

Current therapeutic strategies for AD remain largely symptomatic. Cholinesterase inhibitors, including donepezil, galantamine, and rivastigmine, are widely used to enhance cholinergic neurotransmission and provide modest symptomatic relief, while the N-methyl-D-aspartate (NMDA) receptor antagonist memantine is prescribed for moderate-to-severe stages to attenuate glutamate-mediated excitotoxicity (Marucci et al., 2021; Scarano et al., 2025). More recently, anti-amyloid monoclonal antibodies such as aducanumab, lecanemab, and donanemab have received regulatory approval, representing the first generation of disease-modifying therapies, although their clinical benefit remains modest and is accompanied by risks of amyloid-related imaging abnormalities (ARIA) (Alkhalifa et al., 2025; Yadollahikhales and Rojas, 2023). Beyond these approved agents, emerging strategies under active investigation include AD vaccines (Li W. et al., 2025), tau-targeting antibodies (Wang et al., 2025), anti-inflammatory approaches such as interferon-γ modulation (Li X. et al., 2024), nitric oxide–related pathways (Wang Y. et al., 2024), and novel biomarker-guided precision interventions (Jin et al., 2024). Despite these advances, no therapy to date has been shown to halt or reverse disease progression, underscoring the urgent need to identify novel mechanistic targets.

Against this background, the role of post-translational modifications (PTMs) of proteins in the molecular pathogenesis of AD has garnered increasing attention in recent years (Qin et al., 2024; Libby et al., 2025). Among these, sugar-derived modifications, which directly reflect the nutritional metabolic state and intracellular homeostasis of the organism, have shown a profound association with the onset and progression of AD (Zhao and Lang, 2023). Based on fundamental differences in their catalytic mechanisms, protein sugar modifications are strictly classified into two major categories. The first category is enzymatic glycosylation, which mainly includes N-glycosylation and O-glycosylation (particularly O-GlcNAcylation). This is a physiological process tightly regulated by glycosyltransferases and glycosidases, and it is extensively involved in protein folding, stability, trafficking, and signal transduction (He et al., 2024; Ye et al., 2023). The second category is non-enzymatic glycation, a spontaneous Maillard reaction between reducing sugars and amino acid residues (e.g., lysine and arginine) on proteins. Following a cascade of rearrangements and oxidations, this process ultimately generates irreversible advanced glycation end-products (AGEs) (Krishnamoorthi et al., 2026). In contrast to physiological enzymatic glycosylation, the formation and accumulation of AGEs typically serve as indicators of pathological aging and oxidative stress damage (Rungratanawanich et al., 2021).

For a long time, enzymatic glycosylation and non-enzymatic glycation have conventionally been investigated as two parallel, independent fields in the study of AD pathogenesis (Zhang W. et al., 2024; Kothandan et al., 2024). However, this artificial division may obscure the underlying global metabolic dysregulation characteristic of the AD pathological state. Decreased cerebral glucose metabolism (cerebral glucose hypometabolism) is a core feature in early AD (Ardanaz et al., 2022). Thus, these two seemingly distinct biochemical processes are, in fact, profoundly interconnected within the AD brain. First, they share glucose as a common metabolic precursor (Dozio et al., 2021). Second, and more importantly, they converge on identical pathological substrates (such as tau, APP, and Aβ) and synergistically participate in regulating the activation of neuroinflammation-related receptors (e.g., RAGE) (Krishnamoorthi et al., 2026; Kang et al., 2024). This suggests that, in the context of AD, the dysregulation of enzymatic glycosylation and the accumulation of non-enzymatic AGEs are not isolated events, but rather exhibit a complex interplay and crosstalk.

Therefore, this review aims to overcome the limitations of previous single-perspective approaches by systematically summarizing and integrating the latest research advances regarding protein glycosylation and glycation in AD pathology. We will first elucidate the biochemical foundations of both types of modifications, and subsequently detail their specific regulatory networks in aberrant tau phosphorylation, Aβ generation and metabolism, and the neuroinflammatory cascade. Furthermore, this review will highlight the potential crosstalk between these two modifications during AD progression. Within this unified framework, we will explore the prospects and challenges of a bidirectional strategy—“enhancing protective enzymatic glycosylation while inhibiting detrimental non-enzymatic glycation”—for the development of diagnostic biomarkers and novel therapeutic targets for AD.

## 2 Literature search strategy

To ensure the rigor and reproducibility of this review, a systematic literature search was performed in three databases (PubMed, Web of Science, and Scopus) covering the period from January 2000 to June 2026. The search combined the following keyword groups using Boolean operators: (“Alzheimer's disease” OR “AD” OR “neurodegeneration”) AND (“glycosylation” OR “N-glycosylation” OR “O-GlcNAcylation” OR “O-GalNAc” OR “glycation” OR “advanced glycation end products” OR “AGEs” OR “RAGE” OR “methylglyoxal”).

Inclusion criteria: (i) peer-reviewed original research articles or reviews published in English; (ii) studies addressing molecular mechanisms, biomarkers, or therapeutic strategies related to glycosylation/glycation in AD; (iii) studies in human tissues, validated animal models, or relevant cellular systems.

Exclusion criteria: (i) conference abstracts and non-peer-reviewed preprints; (ii) studies with insufficient methodological detail; (iii) articles unrelated to AD or neurodegeneration. After removing duplicates, an initial pool of approximately 1,280 records was screened by title and abstract, of which 367 underwent full-text review, and 161 were finally included in this synthesis.

## 3 Classification and pathogenesis of Alzheimer's disease

Alzheimer's disease can be classified based on whether its onset is associated with defined genetic mutations and whether it exhibits familial aggregation, into Familial Alzheimer's Disease (FAD) and Sporadic Alzheimer's Disease (SAD). FAD is a rare form of AD, accounting for approximately 1–5% of all cases. The onset age typically ranges from 40 to 65 years, and in some cases may occur as early as 30 years. FAD is primarily inherited in an autosomal dominant manner, with pathogenic mutations mainly found in the amyloid precursor protein (APP) gene, presenilin-1 (PSEN1) gene, and presenilin-2 (PSEN2) gene (Zhang J. et al., 2024). It should be noted that FAD and Early-Onset Alzheimer's Disease (EOAD) are related but not synonymous categories. EOAD is defined by age of onset before 65 years, and includes both FAD and a substantial proportion of sporadic early-onset cases without identified causal mutations (Zhang and Yang, 2026). SAD is the most common form of AD, representing about 95% of all cases. The onset usually occurs after the age of 65, hence also called Late-Onset Alzheimer's Disease (LOAD). Its development is influenced by a combination of genetic risk factors and environmental factors. Recent studies have identified numerous genetic risk loci associated with SAD, involving pathways related to immune response, lipid metabolism, amyloid-β (Aβ) plaques, neurofibrillary tangles (NFTs), and endocytosis; however, many risk loci remain undiscovered (Fedele, 2023; Kepp et al., 2023).

Current research suggests multiple hypotheses regarding the pathogenesis of Alzheimer's disease, including the cholinergic hypothesis, amyloid hypothesis, tau protein hyperphosphorylation, neuroinflammation, oxidative stress, metal ion dyshomeostasis, glutamate excitotoxicity, microbiota-gut-brain axis, and abnormal autophagy (Zhang J. et al., 2024; Twarowski and Herbet, 2023; Kamatham et al., 2024). Table 1 summarizes the key points of each hypothesis. However, to date, no single hypothesis can fully explain the complex pathogenesis of AD (Figure 1).

**Table 1:** Summarizes the key points of each hypothesis.

Pathogenic Hypothesis	Core mechanism	References
Cholinergic hypothesis	Reduced acetylcholine (ACh) levels and cholinergic activity in the AD brain lead to progressive activity in the AD brain lead to progressive degeneration of cholinergic neurons, ultimately causing cognitive dysfunction.	Hampel et al. (2018)
Amyloid hypothesis	Abnormal cleavage of APP by β- and γ-secretases generates neurotoxic Aβ40 and Aβ42 peptides; excessive aggregation of Aβ forms senile plaques, triggering neuronal damage and inflammatory responses.	O'Brien and Wong (2011)
Tau hyperphosphorylation hypothesis	In AD, tau is abnormally hyperphosphorylated, dissociates from microtubules, and aggregates into paired helical filaments (PHFs), which further form NFTs; NFTs disrupt microtubule structure and block neurotransmitter transport, ultimately causing neuronal death.	Liu et al. (2002)
Neuroinflammation hypothesis	Immune-mediated self-destructive processes may occur in the AD brain, with microglia and astrocytes releasing pro-inflammatory factors and generating reactive oxygen species, leading to inflammatory responses and cell death.	Leng and Edison (2021)
Oxidative stress hypothesis	Imbalance between ROS production and clearance in the AD brain results in oxidative damage to lipids, proteins, and nucleic acids, aggravating Aβ fibrillization and tau hyperphosphorylation, and inducing neuronal apoptosis.	Bai et al. (2022)
Metal ion dysregulation hypothesis	Dysregulated metal ion metabolism in AD may promote Aβ fibril aggregation and induce oxidative stress, contributing to cognitive deficits and neuronal death.	Chen et al. (2023)
Autophagy dysfunction hypothesis	Abnormal autophagy pathways, including reduced fusion efficiency of autophagosomes and lysosomes or impaired autophagy initiation/excessive activation, lead to persistent accumulation of pathological proteins (Aβ, tau, etc.), exacerbating neuronal apoptosis.	Zhang et al. (2021)

**Figure 1:**
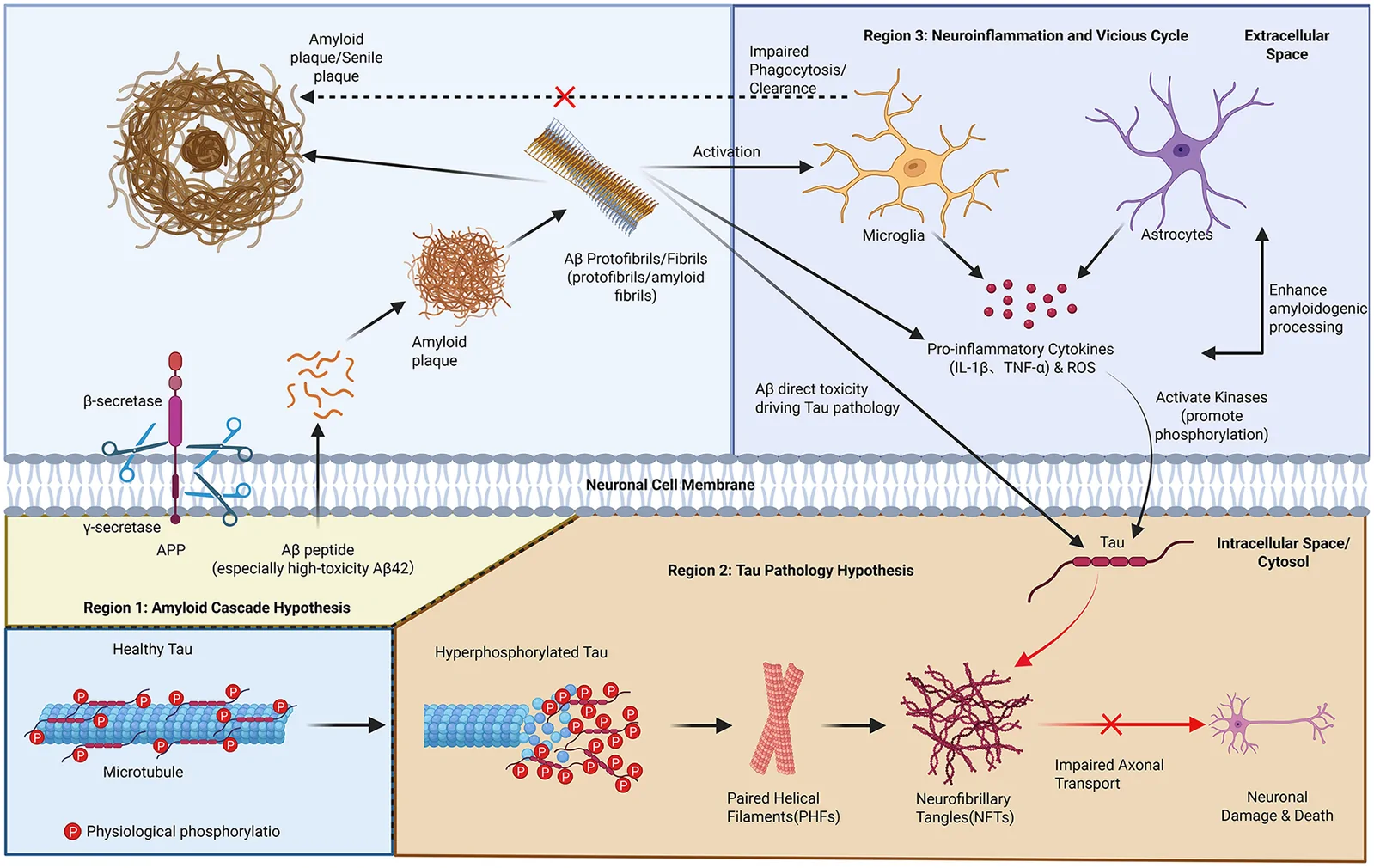
The core pathological cascade and vicious cycle in Alzheimer's disease.

(1) Amyloid Cascade: Transmembrane APP is sequentially cleaved by β- and γ-secretases to release amyloid-β monomers (particularly highly amyloidogenic Aβ). These monomers spontaneously aggregate into highly neurotoxic, soluble oligomers, which further assemble into fibrils structures and immediately deposit outside the cells as senile plaques (featuring a dense amyloid core surrounded by dystrophic neurites). (2) Tau Pathology: Under pathological triggers (e.g., Aβ toxicity and inflammatory milieu), physiologically phosphorylated tau undergoes hyperphosphorylation. This causes tau to detach from microtubules, leading to cytoskeletal collapse. The unbound hyperphosphorylated tau aggregates into paired helical filaments (PHFs) and subsequently forms intracellular neurofibrillary tangles (NFTs). Microtubule breakdown and NFT accumulation collectively impair axonal transport, leading to neuronal death. (3) Neuroinflammation and Vicious Cycle: Soluble Aβ oligomers and fibrils activate microglia and astrocytes. While their physiological Aβ phagocytosis is impaired, these activated glial cells persistently release pro-inflammatory cytokines (e.g., IL-1β, TNF-α) and reactive oxygen species (ROS). Crucially, these inflammatory mediators create a vicious positive feedback loop by further upregulating amyloidogenic APP processing and exacerbating tau hyperphosphorylation, synergistically driving AD progression.

## 4 Overview of protein glycosylation and glycation

Under both physiological and pathological conditions, proteins undergo carbohydrate-mediated modifications via two distinct biochemical routes: glycosylation, an enzyme-regulated post-translational modification (PTM), and glycation, a spontaneous chemical reaction occurring without enzymatic catalysis (Borghi et al., 2025). These two processes differ fundamentally in their underlying mechanisms, the nature of their sugar donors, and their subsequent biological consequences (Table 2, Figure 2).

**Table 2:** Diagram comparing the differences between glycosylation and glycation.

Feature	Glycosylation	Glycation/AGEs
Catalytic mechanism	Enzymatic reaction (catalyzed synergistically by glycosyltransferases and glycosidases)	Non-enzymatic spontaneous chemical reaction (Maillard reaction)
Site of occurrence	Endoplasmic Reticulum (ER), Golgi apparatus (N-glycosylation, classical O-glycosylation); Cytoplasm, Nucleus (O-GlcNAcylation)	Occurs in both intracellular and extracellular environments; significantly accelerated in microenvironments with high glucose and high oxidative stress
Sugar dono	Activated sugar derivatives, including UDP-monosaccharides, GDP-monosaccharides, dolichol pyrophosphate-linked oligosaccharides, etc.	Free reducing sugars (e.g., glucose, fructose), and reactive dicarbonyl compounds generated from glycoxidation and lipid peroxidation
Linkage type	N-glycosidic bond (Asparagine, Asn) or O-glycosidic bond (Serine/Threonine, Ser/Thr)	Non-specific covalent modification, mainly occurring on Lysine (Lys), Arginine (Arg) residues and N-terminal amino groups of proteins; no consensus sequence motif
Reversibility	O-GlcNAcylation is a fully reversible dynamic cycle; the synthesis process of N-glycosylation and classical O-glycosylation can be trimmed by glycosidases, while removal of mature glycans is generally irreversible	Covalent modification and cross-linking of proteins by advanced glycation end products (AGEs) are completely irreversible; only early reaction intermediates (Schiff bases, Amadori products) are reversible
Physiological function	Involved in multiple core physiological processes: protein folding quality control, intracellular trafficking, membrane localization, cell-cell recognition, signal transduction, etc.	Non-specific damaging modification at physiological levels; no clear physiological function identified to date. Accumulates abundantly under pathological conditions and mediates cytotoxicity and tissue damage
Role in alzheimer's disease (AD)	Has a dual role: Downregulation of O-GlcNAcylation promotes hyperphosphorylation and aggregation of tau protein, exerting neurotoxicity; physiological levels of glycosylation maintain normal metabolism of amyloid precursor protein (APP) and neuronal function, exerting neuroprotective effects	Clear neurotoxicity: Promotes abnormal aggregation and deposition of Aβ and tau proteins; mediates chronic neuroinflammation and oxidative stress via the receptor for advanced glycation end products (RAGE), driving AD pathological progression
Therapeutic strategy direction	Upregulate protein glycosylation levels, e.g., increase O-GlcNAcylation via O-GlcNAcase (OGA) inhibitors	Inhibit glycation reactions, scavenge preformed AGEs, and block RAGE-mediated signaling pathways

**Figure 2:**
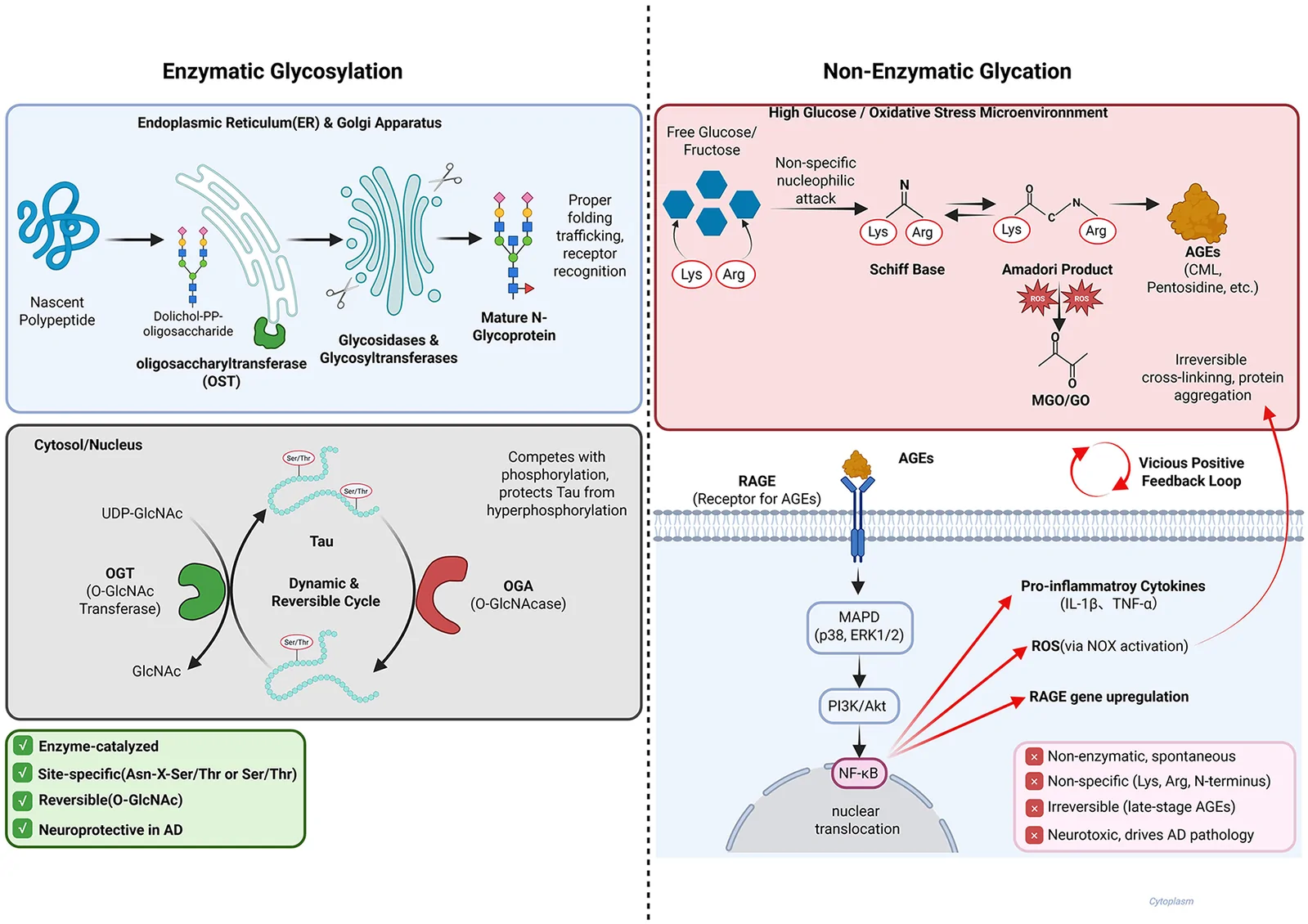
Comparative mechanisms of enzymatic glycosylation and non-enzymatic glycation.

### 4.1 Enzymatic glycosylation

Enzymatic glycosylation represents one of the most prevalent and complex PTMs in eukaryotes and is also widely distributed in prokaryotes (Liu et al., 2010; Mohanty et al., 2020). This process is orchestrated by specific glycosyltransferases and glycosidases, exhibiting high site-specificity and structural diversity of the resulting glycans (He et al., 2024; Togayachi et al., 2026). Based on the linkage between the glycan and the amino acid residue, it is primarily categorized into N-glycosylation and O-glycosylation (Pradeep et al., 2023).

N-glycosylation initiates in the endoplasmic reticulum (ER) and undergoes complex trimming and maturation within the Golgi apparatus (Fisher et al., 2019; Aebi, 2013). It is characterized by the covalent attachment of N-acetylglucosamine (GlcNAc) at the reducing end of the glycan chain to the amide nitrogen of an asparagine (Asn) residue on the target protein (An et al., 2009; Stavenhagen et al., 2019). This attachment primarily occurs at the highly conserved consensus sequence Asn-X-Ser/Thr (where X represents any amino acid except proline). Additionally, certain non-canonical sequence motifs have also been identified as viable sites for N-glycosylation (Mohanty et al., 2020; Garapati et al., 2024). N-glycosylation plays an indispensable role in protein folding quality control, inter-organelle trafficking, membrane localization, and receptor-ligand recognition (Kim and Yoo, 2026).

In contrast to N-glycosylation, the core feature of O-glycosylation is the attachment of glycan chains via O-glycosidic bonds to the side-chain hydroxyl groups of amino acid residues, most commonly serine (Ser) or threonine (Thr) (Ondruskova et al., 2021). While traditional secretory O-glycosylation (e.g., mucin-type) occurs predominantly in the Golgi apparatus (Varki and Kornfeld, 2022), a specialized form of O-glycosylation—O-GlcNAcylation—is of paramount importance in the field of neuroscience (Reily et al., 2019). Occurring within the cytoplasm and nucleus, O-GlcNAcylation involves the dynamic and reversible addition of a single GlcNAc molecule to Ser/Thr residues (Reily et al., 2019; Lubas et al., 1997; Kreppel et al., 1997). This process is uniquely regulated by a single pair of enzymes: O-GlcNAc transferase (OGT), which is responsible for adding the sugar moiety, and O-GlcNAcase (OGA), which facilitates its removal (Reily et al., 2019; Wells et al., 2001). Notably, the substrate sites for O-GlcNAcylation (Ser/Thr) overlap extensively with protein phosphorylation sites. Beyond direct competition for the same site, these two modifications can exert indirect bidirectional regulation through steric hindrance or by inducing protein conformational changes. This dynamic interplay is critical for maintaining the homeostasis of neuronal cytoskeletal proteins, such as tau (Chen et al., 2019; Varki et al., 2022; Mannino and Hart, 2022; Pratt and Vocadlo, 2023).

### 4.2 Non-enzymatic glycation and AGEs

Unlike enzymatic glycosylation, which is strictly regulated by genes and enzymes, non-enzymatic glycation is a spontaneous chemical reaction dependent on concentration and time (Martin et al., 2025). In the oxidative stress microenvironment associated with Alzheimer's disease (AD), this non-enzymatic reaction is abnormally accelerated, becoming a critical mechanism driving irreversible protein damage (Kothandan et al., 2024; Boccardi et al., 2025).

#### 4.2.1 Biochemical cascade of the Maillard reaction

The core of non-enzymatic glycation is the classical Maillard reaction, a process that occurs extremely slowly under physiological conditions but is significantly accelerated under conditions of hyperglycemia or high oxidative stress (Martin et al., 2025). This cascade involves three progressive stages: (1) Early stage: Nucleophilic addition occurs between the free carbonyl groups of reducing sugars (e.g., glucose, fructose) and the ε-amino groups of lysine (Lys), the guanidino groups of arginine (Arg), or the α-amino groups of the protein N-terminus, forming reversible Schiff bases (Krishnamoorthi et al., 2026; Twarda-Clapa et al., 2022). (2) Intermediate stage: Schiff bases undergo slow intramolecular rearrangement to yield relatively stable yet biochemically reversible glycosylated amines: aldose-derived Schiff bases generate Amadori products, while ketose-derived ones form Heyns products (Twarda-Clapa et al., 2022; Lux et al., 2025). (3) Late stage: Over time, driven by continuous oxidation, dehydration, and cleavage, Amadori/Heyns products convert into highly reactive α-dicarbonyl intermediates, such as methylglyoxal (MGO), glyoxal (GO), and 3-deoxyglucosone (3-DG). Additionally, these dicarbonyl compounds are generated in abundance through the auto-oxidation of reducing sugars and lipid peroxidation. These intermediates, possessing strong electrophilicity, rapidly undergo secondary addition and cross-linking with proteins, ultimately resulting in the formation of structurally complex and highly heterogeneous advanced glycation end-products (AGEs) (Peng et al., 2024). Some cross-linking AGEs exhibit autofluorescence, and the vast majority of these covalent modifications are completely irreversible (Twarda-Clapa et al., 2022).

#### 4.2.2 Major characterized AGEs in the CNS

AGEs are not single molecules but a highly heterogeneous collection of compounds (Boccardi et al., 2025). In the central nervous system (CNS), several specific AGEs closely linked to AD pathology have been identified (Kothandan et al., 2024). Among these, Nε-(carboxymethyl)lysine (CML) is the most abundant and extensively studied non-fluorescent AGE in brain tissue, serving as a standard biomarker for evaluating the level of non-enzymatic protein modification *in vivo* (Simm, 2013). Furthermore, pentosidine and pyrraline, which are cross-linking AGEs, are widely detected within senile plaques and neurofibrillary tangles in AD patients (Kothandan et al., 2024; Boccardi et al., 2025). These AGE-mediated covalent modifications and cross-links not only directly induce conformational fixation of substrate proteins (such as tau and Aβ), rendering them highly resistant to proteolytic degradation, but also significantly promote protein misfolding and abnormal aggregation, thereby accelerating the formation and deposition of characteristic AD pathological hallmarks (Krishnamoorthi et al., 2026; Boccardi et al., 2025).

#### 4.2.3 Structural characteristics and ligand recognition of RAGE

The pathological toxicity of AGEs arises not only from direct structural damage to proteins but also from receptor-mediated intracellular signal transduction. The receptor for advanced glycation end-products (RAGE) is a single-transmembrane receptor belonging to the immunoglobulin superfamily (Li H. et al., 2025). Its extracellular region comprises one V-type domain and two C-type domains (C1 and C2), with the positively charged surface of the V-type domain serving as the core region for recognizing and binding AGEs (Dong et al., 2022; Koerich et al., 2023). Notably, RAGE does not only bind to AGEs; it also recognizes other damage-associated molecular patterns (DAMPs) present in the AD pathological microenvironment, including oligomeric Aβ molecules (Kothandan et al., 2024; Li H. et al., 2025). This multi-ligand recognition characteristic positions RAGE as a pivotal “signal amplifier” in AD pathogenesis (Kothandan et al., 2024; Boccardi et al., 2025).

#### 4.2.4 Downstream signaling and pathological positive feedback loop

Upon the binding of AGEs or Aβ to RAGE on the surface of microglia, astrocytes, and neurons, the intracellular domain of the receptor transmits signals via adaptor proteins (such as mDia1) to initiate complex kinase cascades, including the mitogen-activated protein kinase (MAPK) pathways (e.g., p38, ERK1/2) and the PI3K/Akt pathway (Li H. et al., 2025; Bhattacharya et al., 2023; Juranek et al., 2025).

These kinase cascades ultimately activate the IκB kinase (IKK) complex, which mediates the phosphorylation and ubiquitin-dependent degradation of the NF-κB inhibitor IκBα, thereby releasing NF-κB from cytoplasmic sequestration. Simultaneously, the MAPK pathway further phosphorylates the p65 subunit of NF-κB, enhancing its transcriptional activity and promoting its nuclear translocation and target gene transcription (Li H. et al., 2025; Bhattacharya et al., 2023; Saha et al., 2007). In the nucleus, NF-κB upregulates the transcription of various pro-inflammatory cytokines, such as IL-1β, IL-6, and TNF-α, thereby inducing and amplifying chronic neuroinflammation in the CNS (Cross et al., 2024). Furthermore, it enhances the continuous generation of ROS by upregulating the transcriptional expression of NADPH oxidase (NOX) subunits (Kim and Moon, 2024). Moreover, RAGE activation can directly recruit and activate the NOX complex through small GTPases such as Rac1, rapidly inducing high levels of ROS production in a transcription-independent manner (Seryogina et al., 2024). Crucially, NF-κB activation retroactively promotes the expression of RAGE on the target cell membrane. This mechanism establishes an AGEs-RAGE-oxidative stress-inflammation positive feedback loop in the AD brain, leading to sustained elevation of oxidative stress in the microenvironment, which in turn catalyzes the generation of more free AGEs, creating a vicious pathological cycle (Boccardi et al., 2025; Pomroy et al., 2025).

Enzymatic Glycosylation (Left Panel): A highly regulated post-translational modification occurring in the ER/Golgi (N-glycosylation) or cytosol/nucleus (O-GlcNAcylation). Specific glycosyltransferases (e.g., OST, OGT) catalyze the transfer of sugar moieties from activated donors (e.g., UDP-GlcNAc) to defined amino acid residues (Asn-X-Ser/Thr or Ser/Thr). O-GlcNAc modification is dynamically reversible via OGT/OGA cycling and competes with phosphorylation to maintain protein homeostasis, exerting neuroprotective effects in AD.

(Right Panel) Non-Enzymatic Glycation (AGEs Formation): A spontaneous, concentration-dependent Maillard reaction accelerated under hyperglycemia and oxidative stress. Free reducing sugars non-specifically attack nucleophilic residues (Lys, Arg) on proteins, forming reversible Schiff bases and Amadori products, which ultimately undergo oxidation and cross-linking to generate irreversible AGEs (e.g., CML, Pentosidine). AGEs bind to RAGE receptors, activating MAPK/PI3K/NF-κB signaling cascades that drive chronic neuroinflammation, ROS production, and RAGE upregulation, forming a vicious positive feedback loop that exacerbates AD pathology.

## 5 Glycosylation and glycation in AD pathology

### 5.1 Regulation of tau protein by sugar modifications

#### 5.1.1 The reciprocal relationship between O-GlcNAcylation and tau phosphorylation

The physiological function of tau protein is critically dependent on the dynamic equilibrium of its post-translational modification (PTM) status (Alquezar et al., 2020). Research has demonstrated that tau is enriched with serine (Ser) and threonine (Thr) residues. These sites serve as common targets for both kinases, such as glycogen synthase kinase 3β (GSK-3β) and cyclin-dependent kinase 5 (CDK5), and the enzyme O-GlcNAc transferase (OGT) (Hong et al., 2025; Yang et al., 2024; El Hajjar et al., 2024).

These two modifications exhibit a reciprocal relationship characterized by direct competitive occupancy at several key amino acid residues, as well as indirect bidirectional regulation via steric hindrance, protein conformational changes, and the modulation of kinase/phosphatase activities (Zhao et al., 2025; Cantrelle et al., 2021). In the healthy brain, physiological levels of O-GlcNAcylation inhibit the aberrant hyperphosphorylation of tau through site occupancy and conformational stabilization. This maintains the microtubule-binding affinity of tau and exerts a pivotal neuroprotective effect (Alhadidy M et al., 2025; Dasari and Kalyaanamoorthy, 2025).

However, in the AD brain, cerebral glucose hypometabolism and oxidative stress impair the flux of the hexosamine biosynthetic pathway (HBP). This is accompanied by decreased OGT activity and elevated O-GlcNAcase (OGA) activity, ultimately leading to a significant reduction in tau O-GlcNAcylation(Kim et al., 2023; Ran et al., 2025). Once this balance is disrupted, a multitude of Ser/Thr sites previously protected by O-GlcNAc become exposed, leading to uncontrolled tau phosphorylation, its dissociation from microtubules, and subsequent pathological aggregation (Pratt and Vocadlo, 2023; Morales and Pratt, 2024).

#### 5.1.2 Impact of N-glycosylation on tau aggregation

Although tau is classically defined as a cytosolic microtubule-associated protein, its sequence contains conserved consensus sequences (Asn-X-Ser/Thr) for N-glycosylation. Recent studies have indeed detected aberrant N-glycosylation of tau within paired helical filaments (PHFs) extracted from AD brains (Zhao and Lang, 2023; Hong et al., 2025). It is important to clarify that the enzymatic machinery for N-glycosylation resides exclusively within the endoplasmic reticulum (ER) and Golgi apparatus. Under physiological conditions, cytosolic tau cannot undergo this modification.

Although N-linked glycosylation of tau has been reported in AD brain tissue, the underlying mechanism remains incompletely understood (Zhang et al., 2020). Because tau is predominantly a cytosolic protein, its N-glycosylation raises the fundamental question of how it accesses the luminal glycosylation machinery of the endoplasmic reticulum (ER) and Golgi apparatus. One proposed hypothesis is that, under pathological conditions, cytosolic tau may be aberrantly translocated into the ER/Golgi compartments, where it becomes accessible to oligosaccharyltransferases (Zhang et al., 2020; Conroy et al., 2021; Haukedal et al., 2023). However, direct evidence for this trafficking event in AD brain is currently lacking, and the model remains largely speculative. Alternative interpretations should also be considered: current evidence for tau N-glycosylation relies substantially on lectin-based detection and glycosidase sensitivity assays, whose specificity and quantitative reliability in complex brain lysates have inherent limitations. Furthermore, most existing studies have not provided site-resolved data or high-resolution subcellular localization evidence confirming that the glycosylated tau species are physically present within the secretory pathway (Zhang et al., 2020; Sato et al., 2001). Consequently, whether tau N-glycosylation represents a genuine and functionally relevant pathological modification, or partly reflects methodological confounders, remains an open question. Future work employing quantitative glycoproteomic approaches with orthogonal validation will be essential to resolve this issue.

The aberrantly acquired N-glycans on tau significantly alter the protein's local charge, hydrophobicity, and spatial conformation. These changes not only interfere with normal microtubule binding but also drive the transition of tau monomers into pro-aggregatory pathological conformations (Powell et al., 2024, 2025). Furthermore, aberrant N-glycosylation often acts synergistically with hyperphosphorylation, not only accelerating the formation of insoluble neurofibrillary tangles (NFTs) but also promoting the extracellular secretion and prion-like pathological propagation of tau, thereby driving the progression of AD(Kang et al., 2024; Losev et al., 2019).

#### 5.1.3 AGE-modified tau: promotion of aggregation and toxicity

In contrast to tightly regulated enzymatic glycosylation, the formation of advanced glycation end-products (AGEs) via non-enzymatic glycation primarily involves covalent modification of the ε-amino group of lysine (Lys), the guanidino group of arginine (Arg), and the α-amino group of the protein N-terminus (Martin et al., 2025; Uceda et al., 2024). Within the persistent oxidative stress microenvironment of the AD brain, the accumulation of intracellular free reactive dicarbonyl compounds leads to extensive glycation of tau (Yang et al., 2022).

AGE-mediated covalent cross-linking “freezes” tau in its pathological conformation and hinders its ubiquitination, rendering the protein highly resistant to degradation via the ubiquitin-proteasome and autophagy-lysosome pathways (Alquezar et al., 2020; Tangavelou and Bhaskar, 2024). This facilitates the formation of high-molecular-weight insoluble aggregates. Moreover, glycated tau can function as a Damage-Associated Molecular Pattern (DAMP). By binding to RAGE on the surface of neurons and glial cells, it activates intracellular oxidative stress cascades and pro-inflammatory signaling pathways, further exacerbating cytoskeletal collapse and neuronal apoptosis (Rungratanawanich et al., 2021; Cross et al., 2024).

### 5.2 Regulation of Aβ metabolism by sugar modifications

#### 5.2.1 N-/O-glycosylation of APP and secretase cleavage

Amyloid precursor protein (APP) is a prototypical transmembrane glycoprotein. Its N- and O-glycosylation, which occur within the endoplasmic reticulum (ER) and Golgi apparatus, play a critical role in regulating its subcellular localization, protein folding, and the selection of enzymatic cleavage pathways (Kim and Yoo, 2026; Lin et al., 2022). N-glycosylation of the APP extracellular domain is not only essential for its proper folding and vesicular trafficking to the plasma membrane but also modulates its binding affinity for extracellular ligands (Seo et al., 2025; Hao et al., 2025).

More importantly, O-glycosylation sites on APP are concentrated in the flanking regions of the β-site APP cleaving enzyme 1 (BACE1) cleavage site. This modification can directly impede the binding of BACE1 to the APP substrate through steric hindrance, thereby inhibiting the amyloidogenic pathway and shifting APP processing toward the non-amyloidogenic route to generate neuroprotective sAPPα (Vela Navarro et al., 2025; Singh et al., 2021). Abnormalities in the APP glycosylation pattern in the AD brain—such as an increase in high-mannose glycans and a decrease in complex-type glycans—lead to the aberrant sequestration of APP within the acidic environment of endosomes. This significantly increases the probability of its cleavage by BACE1, providing a molecular basis for the shift toward amyloidogenic processing (Kang et al., 2024; Lin et al., 2022).

#### 5.2.2 Glycosylation and enzymatic activity of BACE1 and γ-secretase

The secretase complexes that mediate the amyloidogenic cleavage of APP are themselves strictly regulated by glycosylation during their maturation and activation. BACE1, the core functional subunit of β-secretase, possesses four conserved N-glycosylation sites. Modifications at these sites are indispensable for maintaining protein stability, proper folding, and hydrolytic activity. Specific glycan modifications at these sites preserve the conformational stability of BACE1 within acidic endosomes, while its endosomal subcellular localization is primarily mediated by the dileucine sorting signal on its cytosolic tail (Wen et al., 2022; Liang et al., 2025).

Furthermore, the function of Nicastrin (NCT), a key subunit of the γ-secretase complex, is highly dependent on N-glycosylation. NCT must undergo progressive glycosylation maturation in the ER and Golgi—transitioning from high-mannose to complex-type glycans—to correctly assemble with the PS1, APH-1, and PEN-2 subunits into a functional tetrameric γ-secretase complex. Concurrently, mature N-glycosylation stabilizes the conformation of the NCT extracellular domain, ensuring its ability to recognize APP substrates (Zhao and Lang, 2023; Yu et al., 2025). Consequently, inhibiting the aberrant glycosylation processing of these key secretases has emerged as a potential therapeutic strategy for AD to reduce Aβ production (Urano et al., 2020).

#### 5.2.3 AGE-modified Aβ: enhanced aggregation and neurotoxicity

Glycation of the Aβ peptide is not restricted to the extracellular deposition stage; it can also occur during intracellular generation, where reactive dicarbonyl compounds in the microenvironment attack Aβ to form AGE-modified Aβ (AGE-Aβ) (Krishnamoorthi et al., 2026; Akhter et al., 2025). Compared to native Aβ monomers, AGE-Aβ exhibits a significantly enhanced aggregation propensity, markedly accelerating the fibrillization process and plaque deposition (Sirangelo and Iannuzzi, 2021; Nam et al., 2021).

Moreover, glycated Aβ preferentially binds to RAGE on the surface of glial cells rather than to scavenger receptors that mediate phagocytic clearance. This shift makes it more recalcitrant to degradation by microglia. AGE-Aβ also demonstrates significantly potent neurotoxicity, as it more robustly induces synaptic loss, neuronal dysfunction, and apoptosis by activating oxidative stress and pro-inflammatory signaling pathways (Kang et al., 2024; Akhter et al., 2025; Ayoub et al., 2025).

### 5.3 Sugar modifications in neuroinflammation

#### 5.3.1 Glycosylation changes in microglia

As the resident immune cells of the brain, microglia utilize key surface receptors—such as TREM2 and CD33—that are highly glycosylated transmembrane proteins to mediate pathological recognition, phagocytosis, and activation regulation (Tang et al., 2025). During AD progression, the N-glycosylation of microglia undergoes significant and heterogeneous remodeling (Fastenau et al., 2024; Grijaldo-Alvarez et al., 2024). Historically, microglia have been dichotomously classified into pro-inflammatory (M1) and anti-inflammatory (M2) phenotypes. However, recent single-cell and single-nucleus transcriptomic studies have fundamentally reshaped this view, revealing that microglia in AD exist along a continuum of heterogeneous, disease-associated states rather than discrete polarization endpoints (Chen and Colonna, 2021). Notably, a distinct subpopulation termed disease-associated microglia (DAM) has been identified, characterized by downregulation of homeostatic markers (e.g., P2RY12, TMEM119, CX3CR1) and upregulation of AD-relevant genes including TREM2, APOE, CST7, and LPL. Additional subsets, such as interferon-responsive microglia, lipid-droplet-accumulating microglia (LDAM), and MHC-II–enriched microglia, have also been described (Chen and Colonna, 2021; Gerrits et al., 2021). Given this complexity, the simplistic M1/M2 framework is now widely regarded as inadequate to capture microglial biology in AD (Martins-Ferreira et al., 2025). In the context of glycosylation and glycation, the AGE-RAGE signaling pathway may be involved in the abnormal activation of microglia in AD and the associated neuroinflammation. However, the specific glycomolecular determinants of each subpopulation still need to be further clarified (Cross et al., 2024; Haukedal and Freude K, 2020.

This dynamic alteration in glycosylation patterns directly regulates the membrane stability, ligand-binding affinity, and downstream signal transduction of microglial receptors. Notably, the functional abnormality of CD33, a genetic risk factor for AD, is fundamentally rooted in its aberrant glycosylation, which impairs the microglial phagocytic clearance of Aβ. Conversely, N-glycosylation of TREM2 is a prerequisite for maintaining its stable expression on the cell surface and ensuring its capacity for Aβ phagocytosis and repair (Gonzalez-Gil et al., 2022; Fremuth et al., 2025). Abnormal glycosylation in AD-associated microglia often leads to impaired pathological recognition and clearance functions, locking these cells into a chronically activated state characterized by the sustained release of pro-inflammatory cytokines, thereby driving the progression of AD neuroinflammation (Fastenau et al., 2024).

#### 5.3.2 AGEs-RAGE pathway and NF-κB-Mediated neuroinflammation

The persistent accumulation of AGEs in the extracellular space of the AD brain provides a continuous stream of activation signals for chronic neuroinflammation. Upon the recognition and binding of AGEs to RAGE receptors on the surface of microglia and astrocytes, intracellular MAPK and PI3K/Akt kinase cascades are rapidly activated, subsequently triggering the IκB kinase (IKK) complex. The latter mediates the phosphorylation and ubiquitin-dependent degradation of the NF-κB inhibitor IκBα, releasing NF-κB from cytoplasmic sequestration. Simultaneously, the MAPK pathway further phosphorylates the p65 subunit of NF-κB to enhance its transcriptional activity, ultimately triggering nuclear translocation and target gene transcription (Li H. et al., 2025; Bhattacharya et al., 2023; Yue et al., 2022).

The activation of this transcriptional mechanism not only significantly upregulates the transcription and release of pro-inflammatory cytokines, such as IL-1β, TNF-α, and IL-6, thus amplifying chronic CNS neuroinflammation, but also facilitates further overexpression of RAGE itself via the binding of NF-κB to the RAGE promoter, establishing a positive feedback loop (Boccardi et al., 2025; Koerich et al., 2023; Bhattacharya et al., 2023). Therefore, the inflammatory cascade orchestrated by the AGEs-RAGE axis serves as a critical driver for the sustained expansion of the inflammatory microenvironment and the accelerated pathological progression within the AD brain (Boccardi et al., 2025; Bhattacharya et al., 2023).

### 5.4 Crosstalk between enzymatic glycosylation and non-enzymatic glycation

Although enzymatic glycosylation and non-enzymatic glycation are biochemically distinct processes, they are intricately intertwined within the AD pathological microenvironment. They interact through metabolic pathways, pathological substrates, and signaling networks, forming a complex crosstalk that synergistically drives AD progression.

#### 5.4.1 Competition based on shared metabolic substrates

Cerebral glucose metabolic impairment is one of the earliest pathological hallmarks of AD, and the resulting brain insulin resistance is often referred to as “Type 3 diabetes” (Atabi et al., 2025). The metabolic fate of glucose within brain cells essentially dictates the dynamic equilibrium between these two types of sugar modifications (Bondar et al., 2025). Under physiological conditions, approximately 2%−5% of intracellular glucose enters the hexosamine biosynthetic pathway (HBP) to generate uridine diphosphate N-acetylglucosamine (UDP-GlcNAc). As the central sugar donor for intracellular glycosylation, UDP-GlcNAc not only provides substrates for O-GlcNAc modification but also serves as a critical precursor for N-glycosylation, glycolipids, and proteoglycans, thereby maintaining protein glycosylation homeostasis and normal physiological function (Seo et al., 2025; Paneque et al., 2023).

However, in the AD brain, the significant decline in cerebral glucose uptake and the inhibition of the HBP rate-limiting enzyme, GFAT, by oxidative stress collectively lead to a contracted HBP flux. This results in an insufficient supply of UDP-GlcNAc, causing a comprehensive reduction in protective enzymatic glycosylation, such as O-GlcNAcylation and N-glycosylation (Kang et al., 2024; Kim et al., 2023; Park et al., 2023). Concurrently, persistent oxidative stress and glycolytic dysfunction in the AD brain induce the abnormal accumulation of reactive dicarbonyl compounds, such as methylglyoxal (MGO) generated from the spontaneous degradation of glucose oxidation intermediates and triose phosphates. These compounds spontaneously trigger the Maillard reaction, leading to the massive formation of AGEs (Vašková et al., 2025; Gayger-Dias et al., 2025).

This pathological metabolic shunting, rooted in shared precursors, manifests as an imbalance in the AD brain: the suppression of physiologically protective glycosylation and the proliferation of pathologically damaging glycation (Krishnamoorthi et al., 2026; Uceda et al., 2024). This competitive relationship serves as a primary driver of AD pathogenesis from a metabolic perspective (Boccardi et al., 2025).

#### 5.4.2 Competition and synergy on pathological substrates

Taking tau protein as a central example, these two sugar modifications exert a sequential synergistic effect at the molecular level. Initially, O-GlcNAc modification targets the Ser/Thr residues of tau, competitively inhibiting the phosphorylation of these sites by kinases through site occupancy and conformational stabilization (Dasari and Kalyaanamoorthy, 2025; Barbhuiya et al., 2024). As O-GlcNAc levels decline in the AD brain, these protected sites become vulnerable to aberrant phosphorylation, triggering tau dissociation from microtubules and subsequent conformational collapse (Kim D et al., 2024). Subsequently, the conformationally abnormal tau not only exposes its sequestered Lys and Arg residues but also aggregates in the cytoplasm, becoming highly susceptible to attack by reactive dicarbonyl compounds (Ayoub et al., 2025; Li W. et al., 2024). Finally, AGEs “lock” tau into its pathological conformation via irreversible covalent cross-linking, resulting in the formation of insoluble NFTs (Boccardi et al., 2025; Alrouji et al., 2025).

Similarly, the generation and pathological toxicity of Aβ are synergistically regulated by both modifications. The N- and O-glycosylation patterns of APP influence the selection of its cleavage pathways, thereby determining the rate of Aβ production. Once Aβ is generated and deposited extracellularly, AGE modifications rapidly accelerate its fibrillization, significantly enhancing its neurotoxicity and pro-inflammatory effects (Zhao and Lang, 2023; Kang et al., 2024; Akhter et al., 2025; Tachida et al., 2023). This sequential “multi-hit” mechanism on the same pathological substrate drastically accelerates the disintegration of protein homeostasis and the deposition of pathological hallmarks in the AD brain (Kothandan et al., 2024; Yu et al., 2025).

#### 5.4.3 Convergence of neuroinflammatory pathways

At the level of cellular signaling, these two sugar modifications converge to amplify inflammatory signals and create a vicious cycle through the regulation of glial cell activation. On one hand, abnormal N-glycosylation of critical microglial immune receptors (e.g., TREM2, CD33) directly impairs their ability to recognize and clear Aβ, leading to the sustained accumulation of Aβ and AGEs in the brain. On the other hand, accumulated AGEs bind to the RAGE receptor on glial cells, activating the NF-κB-mediated pro-inflammatory signaling pathway and inducing the release of abundant pro-inflammatory cytokines, thereby establishing a persistent chronic neuroinflammatory microenvironment (Boccardi et al., 2025; Fremuth et al., 2025; Cheng et al., 2025).

Crucially, the reactive oxygen species (ROS) generated in this inflammatory microenvironment not only accelerate non-enzymatic glycation—further increasing AGE production—but also inhibit the catalytic activity of OGT, which exacerbates the decline in O-GlcNAc levels and worsens enzymatic glycosylation abnormalities (Kang et al., 2024; Chen et al., 2019; Boccardi et al., 2025). This process constitutes a complete vicious positive feedback loop: Impairment of enzymatic glycosylation → impaired pathological recognition and clearance → accumulation of Aβ/AGEs → activation of RAGE-NF-κB inflammatory pathway → exacerbated oxidative stress → increased AGE generation + worsened enzymatic glycosylation (Koerich et al., 2023; Cheng et al., 2025). This cross-pathway signaling interplay ensures that the chronic inflammatory response in the AD brain remains in a state of irreversible, sustained activation, acting as a core amplifier that drives AD progression (Bhattacharya et al., 2023; Cheng et al., 2025).

## 6 Diagnostic and therapeutic potential

Given the intricately intertwined pathological network between aberrant enzymatic glycosylation and advanced glycation end products (AGEs) in the pathogenesis of Alzheimer's disease (AD), targeting the molecular features of protein sugar modifications not only provides a novel source of biomarkers for the early diagnosis of AD but also opens up important avenues for developing multi-dimensional intervention strategies.

### 6.1 Glycosylation-related biomarkers

Pathological changes in AD initiate 10–20 years before the onset of clinical cognitive decline. Identifying highly sensitive and specific early biomarkers remains a major challenge for AD clinical translation (Jia et al., 2024; De Strooper and Zetterberg, 2026). With the rapid advancement of high-throughput glycoproteomics technologies, glycan modification features in body fluids are emerging as highly promising tools for AD diagnosis and disease progression monitoring (Chen et al., 2021).

Caveats regarding the current strength of biomarker evidence. The clinical utility of glycan- and AGE-based biomarkers in AD depends critically on the quality of the underlying evidence. As summarized in Table 3, the currently available candidate biomarkers vary considerably in sample type, cohort size, degree of independent replication, and diagnostic specificity relative to other conditions. Most peripheral AGE and glycan alterations reported to date have been derived from single-center studies with modest sample sizes, and several of these changes overlap with those observed in type 2 diabetes and chronic kidney disease, thereby limiting their disease specificity(Li H. et al., 2025; Yamaguchi and Yamaguchi, 2025). On this basis, peripheral AGE and glycan alterations should currently be regarded as candidate biomarkers rather than validated AD-specific markers. Before these candidates can be translated into clinical practice, multi-center prospective studies with harmonized analytical protocols and independent replication cohorts are needed. Furthermore, their diagnostic performance must be directly benchmarked against the established core AD biomarkers [Aβ42/40, p-tau181, p-tau217, GFAP, NfL(Hansson et al., 2022)] to demonstrate added clinical value.

**Table 3:** Summary of current evidence for glycan- and AGE-based candidate biomarkers in AD.

Candidate biomarker	Sample type	Reported change in AD	Cohort size (representative studies)	Independent replication	Diagnostic specificity vs. Other conditions	Current evidence level	Reference
CSF N-glycome (site-specific)	CSF	Altered branching and sialylation patterns	Small–moderate (typically < 200)	Limited	Overlap with other neurodegenerative disorders reported	Exploratory	Chen et al. (2021)
Plasma/serum N-glycome	Plasma/Serum	Increased bisecting GlcNAc, altered sialylation/fucosylation	Moderate (100–500)	Partial replication in independent cohorts	Overlaps with aging and metabolic disease	Preliminary	Zhou et al. (2025)
Serum/plasma AGEs (CML, pentosidine)	Serum/Plasma	Elevated	Small–moderate	Inconsistent across studies	Non-specific; markedly elevated in T2DM and CKD	Non-specific candidate	Rungratanawanich et al. (2021) Kothandan et al. (2024)
CSF AGEs	CSF	Elevated	Small (mostly < 100)	Very limited	Better AD relevance than peripheral AGEs, but insufficient data	Exploratory	Kothandan et al. (2024)
Soluble RAGE (sRAGE)	Plasma/Serum	Decreased	Moderate	Partial	Also altered in cardiovascular and metabolic disease	Non-specific candidate	Rungratanawanich et al. (2021)
Brain-tissue glycan/AGE signatures	Post-mortem brain	Consistently altered	Small (autopsy-limited)	Confirmed in multiple studies	AD-relevant, but not usable for antemortem diagnosis	Mechanistic evidence	Fastenau et al. (2024)

CSF, cerebrospinal fluid; CML, N-ε-carboxymethyllysine; T2DM, type 2 diabetes mellitus; CKD, chronic kidney disease; RAGE, receptor for advanced glycation end products; GlcNAc, N-acetylglucosamine.

Evidence levels are defined as: Mechanistic evidence —reproducible findings in AD brain tissue with clear pathological relevance but no antemortem applicability; Preliminary—reproducible findings in patient biofluids from more than one cohort, with partial independent replication; Exploratory—findings from single or few small studies without independent replication; Non-specific candidate—reproducible changes reported in AD but with substantial overlap with other diseases.

#### 6.1.1 Enzymatic glycosylation-related biomarkers

In both cerebrospinal fluid (CSF) and peripheral blood, AD patients exhibit characteristic abnormalities in N-glycosylation patterns, which correlate closely with AD pathological progression (Zhou et al., 2025). For example, reduced terminal sialylation and abnormal levels of bisecting N-acetylglucosamine (bisecting GlcNAc) on proteins such as amyloid precursor protein (APP), apolipoprotein E (ApoE), and transferrin in the CSF are significantly correlated with cerebral Aβ pathological burden, tau phosphorylation levels, and cognitive scores, making them potential early diagnostic biomarkers for the prodromal stage of AD (Schedin-Weiss et al., 2020; Zhou et al., 2024; Flowers et al., 2020; Hoshi et al., 2021). Furthermore, alterations in the N-glycosylation profile of immunoglobulin G (IgG) in peripheral blood have also been confirmed to correlate with AD risk and the rate of cognitive decline, providing potential targets for non-invasive screening (Zhang W. et al., 2024).

#### 6.1.2 Glycation (AGEs)-related biomarkers

As key products of non-enzymatic glycation, specific AGE molecules can serve as monitoring biomarkers for oxidative stress and metabolic damage in AD (Zhang et al., 2025). The abnormal elevation of characteristic AGE levels—such as Nε-(carboxymethyl)lysine (CML) and pentosidine—in serum and CSF indirectly reflects the oxidative stress and glycation burden in the central nervous system (CNS) and systemically. These levels show significant correlation with AD Braak pathological staging and the severity of cognitive decline (Grosskopf et al., 2024; Dakterzada et al., 2023). Additionally, non-invasive detection of skin autofluorescence, which primarily reflects the accumulation of fluorescent AGEs in skin tissue, has been confirmed by multiple observational studies to be significantly negatively correlated with cognitive decline in AD patients. This technique could serve as a non-invasive surrogate biomarker for systemic AGE accumulation; however, it must be clarified that this metric does not directly reflect central AGE levels and currently serves only as an auxiliary evaluation indicator, having not yet been incorporated into clinical diagnostic guidelines for AD (Mooldijk et al., 2024; Tanito and Koyama, 2025; Palmqvist et al., 2025; Rajendran and Krishnan, 2024).

In summary, integrating specific changes in enzymatic glycan structures with AGE metabolite profiles to construct multi-dimensional, multi-target biomarker panels for early AD screening and disease progression monitoring represents a crucial direction for the future of AD fluid-based diagnostics.

### 6.2 Therapeutic strategies

Given the pathological imbalance in the AD brain—characterized by a decline in protective enzymatic glycosylation and an elevation in damaging glycation—future AD drug development must focus on bidirectional interventions: enhancing physiologically protective enzymatic glycosylation signaling while simultaneously blocking the damaging glycation cascade and its downstream pathological effects (Tables 4, 5).

**Table 4:** Therapeutic strategies targeting protein glycosylation and glycation in Alzheimer's disease.

Enhance protective modifications	Inhibit damaging modifications
OGA inhibitors (e.g., ASN90, MK-8719) → increase cerebral O-GlcNAc levels on proteins → inhibit tau hyperphosphorylation and aggregation	AGEs formation inhibitors (carbonyl scavengers) → reduce de novo formation of AGEs
Improve cerebral glucose metabolism and upregulate the flux of the Hexosamine Biosynthesis Pathway (HBP) → indirectly enhance physiological glycosylation modifications	AGEs cross-link breakers → reverse preformed protein cross-links
Correct aberrant N-glycosylation processing of APP, secretases and immune receptors → restore normal physiological functions of proteins	RAGE antagonists → block the downstream inflammatory and oxidative stress cascades mediated by the AGEs-RAGE axis
Antioxidant strategies → protect the activity of glycosylation-related enzymes such as O-GlcNAc Transferase (OGT)	Antioxidant strategies → reduce the generation of reactive carbonyl precursors for glycation reactions
Combination strategy: OGA inhibitors + RAGE antagonists/AGEs inhibitors → synergistically reshape glycan modification homeostasis and exert bidirectional intervention effects (no clinical validation to date)	

**Table 5:** Clinical translation efforts targeting glycosylation and glycation pathways in AD.

Compound	Target/mechanism	Highest phase	Sponsor	Key endpoints	Outcome/status	Reference
MK-8719	OGA inhibitor (O-GlcNAc↑)	Phase I	Alectos	Safety, PK, target engagement	Completed; favorable safety; development transitioned	Selnick et al. (2019)
Ceperognastat (LY3372689)	OGA inhibitor	Phase II (failed)	Eli Lilly	ADAS-Cog13, iADRS, CDR-SB	Did not meet primary efficacy endpoints; development discontinued	Meade et al. (2026)
ASN51	OGA inhibitor	Phase II	Asceneuron	Safety, target engagement (PET), cognition	Ongoing; central target engagement demonstrated	Sun et al. (2025)
Thiamet-G	OGA inhibitor	Preclinical only	Academic	Tau phosphorylation in mouse models	Positive preclinical; no clinical trials	Yuzwa et al. (2012)
Azeliragon (TTP488)	RAGE antagonist	Phase III (failed)	vTv Therapeutics	ADAS-Cog, CDR-SB	Failed in overall AD population; ongoing subgroup trials in AD+T2DM	Burstein et al. (2018)
Pyridoxamine	Dicarbonyl scavenger, AGE inhibitor	Phase II (diabetic nephropathy)	NephroGenex	AGE reduction	Approved indication is not AD; limited AD-specific data	Dwyer et al. (2015)
Aminoguanidine	AGE inhibitor	Discontinued (nephropathy trial)	Alteon	AGE, renal endpoints	Discontinued due to safety; no AD trials	Bolton et al. (2004)
Alagebrium (ALT-711)	AGE crosslink breaker	Phase II (cardiovascular)	Alteon	Arterial stiffness	Not developed for AD	Hartog et al. (2011)

OGA, O-GlcNAcase; PK, Pharmacokinetics; RAGE, receptor for advanced glycation end products; T2DM, type 2 diabetes mellitus; ADAS-Cog, Alzheimer's Disease Assessment Scale–Cognitive; CDR-SB, Clinical Dementia Rating–Sum of Boxes; iADRS, integrated Alzheimer's Disease Rating Scale; PET, positron emission tomography.

#### 6.2.1 Enhancing protective glycosylation (OGA inhibitors, etc.)

Targeting the pathological mechanism whereby reduced O-GlcNAc levels in the AD brain lead to tau hyperphosphorylation and aggregation, pharmacologically elevating global brain O-GlcNAcylation is currently one of the most promising therapeutic strategies (Park et al., 2020; Wani et al., 2017). Because O-GlcNAc transferase (OGT) is involved in numerous essential intracellular physiological processes, direct activation of OGT is prone to inducing severe off-target toxicity. Therefore, current research and development focus primarily on O-GlcNAcase (OGA) inhibitors—specifically elevating the O-GlcNAc modification of brain proteins by inhibiting OGA's hydrolysis of O-GlcNAc (Kielbasa et al., 2024).

Among therapeutic strategies aiming to enhance protective O-GlcNAcylation, pharmacological inhibition of OGA has attracted the greatest attention. Preclinical studies using tool compounds such as Thiamet-G have consistently demonstrated that elevating brain O-GlcNAc reduces tau hyperphosphorylation, ameliorates neurofibrillary pathology, and improves cognitive performance in mouse models (Pratt and Vocadlo, 2023; Rostgaard et al., 2023). Building on this rationale, several OGA inhibitors have advanced into human trials. MK-8719 (Merck/Alectos) completed Phase 1 with acceptable safety and demonstrated central target engagement. LY3372689 (ceperognastat), developed by Eli Lilly, progressed further and achieved robust central OGA occupancy as confirmed by PET imaging (Kielbasa et al., 2024; Meade et al., 2026; Pokorny et al., 2026).

However, it is essential to distinguish target engagement from clinical disease modification. Eli Lilly announced that ceperognastat failed to meet its primary efficacy endpoints in Phase 2 trials in early symptomatic AD, and its development was subsequently discontinued (Meade et al., 2026; Fleisher et al., 2026). This outcome represents an important cautionary lesson: robust preclinical efficacy and demonstrated target engagement do not necessarily translate into clinical benefit (Fleisher et al., 2026). Various possible explanations have been proposed in the current literature, including the possibility that OGA inhibition may be initiated too late in the disease course, that compensatory mechanisms limit the sustained effect on tau, or that O-GlcNAc regulation is more compartmentalized than currently appreciated (Meade et al., 2026; Shen et al., 2025). Ongoing Phase 1/2 studies of Asceneuron's OGA inhibitors (e.g., ASN51) will provide additional insight, but as of now, no OGA inhibitor has demonstrated cognitive benefit in AD patients (Shen et al., 2025). Similarly, RAGE antagonism via azeliragon failed in Phase 3 trials in unselected AD populations, although exploratory analyses suggest potential efficacy in AD patients with comorbid type 2 diabetes (Meade et al., 2026; Shen et al., 2025). This hypothesis is currently still under further investigation.

Beyond directly targeting OGA, indirect strategies to enhance protective glycosylation are also crucial. These include improving cerebral glucose metabolism and upregulating hexosamine biosynthetic pathway (HBP) flux to increase UDP-GlcNAc supply and protein O-GlcNAcylation, as well as correcting the aberrant N-glycosylation processing of APP, secretases, and immune receptors (Kim et al., 2023; Yu et al., 2025).

#### 6.2.2 Blocking the damaging glycation cascade (AGE inhibitors, RAGE antagonists)

Interventions targeting non-enzymatic glycation and its downstream pathways are primarily divided into three levels: (1) inhibiting AGE formation at the source, (2) breaking existing AGE cross-links, and (3) blocking AGE-RAGE-mediated downstream pathological signaling.

Regarding the inhibition of AGE generation, carbonyl scavengers such as Aminoguanidine and Pyridoxamine can neutralize reactive dicarbonyl intermediates, thereby blocking the Maillard reaction and AGE formation at the source. However, both have failed to meet primary clinical endpoints due to severe adverse effects in clinical trials (Boccardi et al., 2025; Li H. et al., 2025; Koerich et al., 2023). For established pathological AGE cross-links, the cross-link breaker Alagebrium (ALT-711) has demonstrated the ability to reduce tissue stiffness in animal models of diabetic vasculopathy and, to some extent, reduce amyloid deposition in AD models. Nevertheless, there is currently insufficient clinical evidence to support its therapeutic value in AD (Kothandan et al., 2024; Boccardi et al., 2025; Koerich et al., 2023). Furthermore, Azeliragon (TTP488), a small-molecule antagonist targeting the RAGE receptor, can competitively block the binding of ligands (such as AGEs and Aβ) to RAGE, thereby inhibiting the downstream NF-κB inflammatory cascade (Li H. et al., 2025; Koerich et al., 2023). Although this drug showed a trend toward cognitive protection in Phase II clinical trials, it failed to meet the primary clinical endpoint of cognitive improvement in patients with mild-to-moderate AD in Phase III trials (Boccardi et al., 2025; Li H. et al., 2025; Wu et al., 2025). Despite these setbacks, RAGE-targeted therapy remains an important research direction in AD neuroinflammation intervention. This can be advanced by optimizing clinical trial designs and implementing precise pathological stratification of AD patients (e.g., targeting cohorts with high glycation burden and high inflammatory phenotypes) (Boccardi et al., 2025; Li H. et al., 2025; Wu et al., 2025).

Given these clinical realities, combined therapeutic strategies targeting both OGA and RAGE, or anti-glycosylation and anti-glycation methods, remain conceptually attractive but entirely hypothetical at present, with no clinical validation available. Any translational recommendation from this pathway should therefore be interpreted with appropriate caution.

### 6.3 Unresolved questions and future directions

Although research on sugar modification-based AD diagnostics and therapeutics has made substantial progress, the field still faces several critical scientific questions that urgently need to be addressed. First, most current studies focus on single types of modifications in isolation, lacking a systematic characterization of the temporal and spatial heterogeneity of the dynamic crosstalk between enzymatic glycosylation and glycation in the living AD brain (Krishnamoorthi et al., 2026; Ayoub et al., 2025). Future efforts should develop positron emission tomography (PET) tracers with higher specificity to enable real-time, non-invasive monitoring of AGE accumulation and OGA activity *in vivo* (Cook et al., 2023; Zhao and Liang S, 2025).

Second, systematically altering the glycosylation network inevitably introduces off-target effects and narrows safety margins (Meade et al., 2026). For instance, while elevating brain O-GlcNAc levels, OGA inhibitors broadly affect protein O-GlcNAcylation in peripheral tissues. Whether long-term use interferes with normal physiological processes, such as glucose metabolism and insulin signaling, requires more rigorous, long-term toxicological evaluation. Additionally, a core translational bottleneck is how to achieve precise central nervous system (CNS) delivery of anti-glycation and anti-RAGE strategies to inhibit pathological damage without disrupting systemic redox homeostasis (Flax et al., 2020; de Koning et al., 2025).

Third, technical bottlenecks remain in the clinical translation of fluid-based sugar modification biomarkers. Current glycoproteomic technologies still have limited sensitivity for detecting low-abundance glycosylation modifications in body fluids. Furthermore, the correlation and specificity between peripheral blood sugar modification changes and central pathological alterations require validation in large-sample, multicenter clinical cohorts. Establishing standardized detection protocols and determining unified clinical reference thresholds are essential prerequisites for their clinical application (Chen et al., 2021; Zhou et al., 2025, 2024; Wang L. et al., 2024; Zeng et al., 2024).

Finally, given the profound complexity of AD pathogenesis, single-target drugs are highly prone to failure in late-stage clinical trials (Gong et al., 2022; Yiannopoulou et al., 2019). The future therapeutic paradigm will likely need to shift toward combinatorial targeting strategies. For example, guided by precision medicine stratification, combining OGA inhibitors (to protect tau homeostasis) with RAGE antagonists or AGE inhibitors (to quell glia-mediated neuroinflammation) could synergistically remodel the central glucose metabolism and modification networks. This approach ultimately aims to achieve the clinical goal of delaying or even reversing AD pathological progression (Chen et al., 2019; Boccardi et al., 2025; Li H. et al., 2025; Cheng et al., 2025; Djurovic-Topalovic et al., 2025).

## 7 Conclusions and perspectives

This review has integrated enzymatic glycosylation and non-enzymatic glycation within the unifying framework of cerebral glucose hypometabolism in AD, highlighting their convergent impact on tau pathology, APP/Aβ processing, and neuroinflammation. To aid interpretation, we summarize below what is currently established versus speculative in this rapidly evolving field.

**Established findings include:** (i) brain O-GlcNAcylation is globally reduced in AD and reciprocally regulates tau phosphorylation; (ii) AGE accumulation is elevated in AD brain and cerebrospinal fluid; (iii) RAGE signaling mediates AGE- and Aβ-induced neuroinflammatory responses; (iv) cerebral glucose hypometabolism is a well-documented early feature of AD; and (v) altered N-glycan profiles are detectable in AD patient samples.

**Speculative or preliminary areas requiring further validation include:** (i) the precise mechanism and site-specificity of tau N-glycosylation; (ii) the direction of causality between glycation/glycosylation changes and AD pathogenesis versus their occurrence as downstream consequences; (iii) the AD specificity of peripheral glycan and AGE biomarkers; (iv) the clinical translatability of OGA inhibition, in light of the recent Phase 2 failure of ceperognastat; and (v) the therapeutic potential of combined OGA–RAGE or multi-target anti-glycation strategies, which remain entirely hypothetical.

**Future research should prioritize:** (i) site-specific glycoproteomics with rigorous analytical controls; (ii) longitudinal biomarker studies with independent replication; (iii) re-examination of failed clinical trials to identify patient subgroups most likely to benefit; and (iv) integration of glycobiology with single-cell transcriptomics to define disease-associated microglial states.

Taken together, a cautious yet mechanistically informed approach will be essential to translate glycobiological insights into effective disease-modifying therapies for AD. The future therapeutic paradigm will likely need to shift toward combinatorial targeting strategies guided by precision medicine stratification, ultimately aiming to remodel cerebral glucose metabolism and modification networks to delay or even reverse AD pathological progression.
